# Metagenomics reveals the structure of *Mastrevirus*–host interaction network within an agro-ecosystem

**DOI:** 10.1093/ve/vead043

**Published:** 2023-07-06

**Authors:** Sohini Claverie, Murielle Hoareau, Sélim Ben Chéhida, Denis Filloux, Arvind Varsani, Philippe Roumagnac, Darren P Martin, Jean-Michel Lett, Pierre Lefeuvre

**Affiliations:** CIRAD, UMR PVBMT, F-97410 St Pierre, La Réunion, France; Université de La Réunion, UMR PVBMT, F-97410 St Pierre, La Réunion, France; CIRAD, UMR PVBMT, F-97410 St Pierre, La Réunion, France; CIRAD, UMR PVBMT, F-97410 St Pierre, La Réunion, France; CIRAD, UMR PHIM, Montpellier F-34090, France; PHIM Plant Health Institute, Université de Montpellier, CIRAD, INRAE, Institut Agro, IRD, Montpellier 34090, France; CIRAD, UMR PHIM, Montpellier F-34090, France; PHIM Plant Health Institute, Université de Montpellier, CIRAD, INRAE, Institut Agro, IRD, Montpellier 34090, France; Computational Biology Division, Department of Integrative Biomedical Sciences, Institute of Infectious Diseases and Molecular Medicine, University of Cape Town, Observatory 7925, South Africa; CIRAD, UMR PVBMT, F-97410 St Pierre, La Réunion, France; CIRAD, UMR PVBMT, F-97410 St Pierre, La Réunion, France; The Biodesign Center for Fundamental and Applied Microbiomics, Center for Evolution and Medicine, School of Life Sciences, Arizona State University, Tempe, AZ 85287, USA; Structural Biology Research Unit, Department of Integrative Biomedical Sciences, University of Cape Town, Rondebosch, Cape Town 7700, South Africa

**Keywords:** CRESS DNA viruses, viral ecology, host range, recombination

## Abstract

As highly pervasive parasites that sometimes cause disease, viruses are likely major components of all natural ecosystems. An important step towards both understanding the precise ecological roles of viruses and determining how natural communities of viral species are assembled and evolve is obtaining full descriptions of viral diversity and distributions at ecosystem scales. Here, we focused on obtaining such ‘community-scale’ data for viruses in a single genus. We chose the genus *Mastrevirus* (family Geminiviridae), members of which have predominantly been found infecting uncultivated grasses (family Poaceae) throughout the tropical and sub-tropical regions of the world. We sampled over 3 years, 2,884 individual Poaceae plants belonging to thirty different species within a 2-ha plot which included cultivated and uncultivated areas on the island of Reunion. Mastreviruses were found in ∼8 per cent of the samples, of which 96 per cent did not have any discernible disease symptoms. The multitude of host–virus associations that we uncovered reveals both the plant species that most commonly host mastreviruses and the mastrevirus species (such as *maize streak virus* and *maize streak Reunion virus*) that have especially large host ranges. Our findings are consistent with the hypothesis that perennial plant species capable of hosting years-long mixed mastrevirus infections likely play a disproportionately important role in the generation of inter-species and inter-strain mastrevirus recombinants.

## Introduction

It is becoming increasingly apparent that viruses are important components of global ecosystems and that the ecological context of viruses underlies their propensities to switch hosts and emerge as novel pathogens of humans and our domesticated plants and animals (Muthukumar et al. 2009; Roossinck et al. 2010; French and Holmes 2020; Holmes 2022). This realisation has been mostly driven by discoveries made using metaviromics: metagenomic studies exploring viral diversity within a broad range of different host and vector species (Stobbe and Roossinck 2014; Shi, Zhang, and Holmes 2018; Roux, Matthijnssens, and Dutilh 2021). Besides revealing the ubiquity and abundance of viruses, these studies have revealed that the genetic diversity of viruses dwarfs that previously discovered using conventional virus discovery methods (Shi et al. 2016). All these discoveries have firmly emphasised both that a holistic approach is necessary to understand virus emergence (Malmstrom, Melcher, and Bosque-Pérez 2011; Vayssier-Taussat et al. 2014; Shates et al. 2019) and that only a very small fraction of plant–virus interactions manifest as diseases (Roossinck 2011; McLeish et al. 2022).

The pressing need to understand the interplay between the diversity of hosts and that of the viruses that routinely infect them has promoted several ecosystem-scale plant–virus diversity studies (Muthukumar et al. 2009; Roosinck *et al.*, 2010). In particular, agro-ecosystems, where natural environmental borders extensively managed farmlands, were soon identified as ideal settings (Bernardo et al. 2018; García-Arenal and Zerbini 2019; McLeish et al. 2022). Such settings are expected to provide opportunities for novel virus–host interactions that will potentially alter the equilibria of otherwise long-evolved plant–virus interaction networks (Malmstrom et al. 2005; Alexander et al. 2014; Bernardo et al. 2018). These environments have therefore been proposed as natural laboratories to test the interplay between the diversity and distributions of plant hosts and their viruses (Alexander et al. 2014). Yet, the actual structures of even individual viral communities—groups of virus species that epidemiologically and evolutionarily interact with one another—are still largely unknown. This is because the breadth of viral diversity within any given ecosystem is simply too great for it to be fully characterised using currently available research tools. Even when virus-derived genomic sequences are sequenced during metaviromics studies, many of these sequences cannot even be definitively determined to be of viral origin, simply because homologous sequences have never been found before in any previously characterised viruses.

Rather than attempting to analyse all viral diversity within a given environment, an alternative is to focus on the diversity of a single clearly defined group of well-characterised viruses. One such group is the *Mastrevirus* genus of the family Geminiviridae. The most well-known mastrevirus, maize streak virus (MSV), is the causal agent of maize streak disease (MSD): a disease that, over the last century, has periodically devastated maize harvests in most African countries (Shepherd et al. 2010). Mastreviruses are single-stranded circular DNA viruses with ∼2.7-kb genomes. The genus is mostly comprised of monocotyledonous plant-infecting species but also includes some species that infect dicotyledonous plants. Presently, a total of forty-five different mastrevirus species have been characterised, including twenty-three species that were initially identified on cultivated plants and twenty-one species that have only ever been found infecting uncultivated plants. Nineteen of the mastrevirus species that infect monocotyledonous plants have been identified in Africa or on the adjacent Islands of the South West Indian Ocean (SWIO) and these have been collectively called African streak viruses (AfSVs; Kraberger et al. 2017; International Committee on Taxonomy of Viruses, https://ictv.global/taxonomy).

Despite the relatively large number of AfSV genomic sequences that are available in public databases, our understanding of their host ranges is profoundly biased towards crop species. This makes it difficult to actually assess whether the currently known host ranges of AfSVs are the authentic realisation of their infectious capacities or are merely the imprints of host availability or sampling biases in the regions where particular virus species have been studied. In Reunion, where several AfSV species have been identified so far (eight of the nineteen described species), it was demonstrated that most existed in sympatry at the scale of a single field (Kraberger et al. 2017; Claverie et al. 2019).

Here, we describe more precisely the natural host ranges and prevalence of mastreviruses at the scale of this same single field in Reunion and additionally establish the structure of host–virus associations. Besides identifying substantial differences in the prevalence and distributions among hosts of the mastrevirus species, we also identify the host species that are most likely to accommodate mixed mastrevirus infections and which are, therefore, most likely to foster the emergence of novel recombinant mastreviruses.

## Materials and methods

### Sampling

Samples were collected in an agro-ecosystem of ∼2 ha (∼17,000 m^2^) at the Bassin Plat Cirad experimental facility (latitude −21.3231; longitude 55.4912) in Saint-Pierre (Reunion). This agro-ecosystem is a mixed environment containing grassland, wasteland, fallow, and agricultural plots. Leaf samples of monocotyledonous Poales species (cultivated and uncultivated) were randomly collected regardless of their health status (with or without apparent disease symptoms) or sizes during four sampling campaigns in November 2014 (*N* = 144), November 2016 (*N* = 1,196), April 2017 (*N* = 744), and November 2017 (*N* = 800) (see Table 1 for details). A total of 2,884 leaf samples, including thirty plant species from twenty-five genera, were collected. Of the 2,884 samples, 115 samples belonging to eight different species presented with visible symptoms typical of streak disease. Unless directly treated using the ‘leaf soak’ nucleic acid extraction approach (see the “Total genomic DNA extraction” paragraph), all samples were dried in an oven at 50°C overnight and stored at room temperature before use. Plant life-trait histories and origins (such as their crop status, life cycle, and invasiveness) were determined using the Conservatoire Botanique National de Mascarin botanical database (https://mascarine.cbnm.org/).

**Table 1.** A summary of sampled *Poaceae* species for each campaign. The number in brackets refers to the number of samples showing typical streak symptoms.

Tribe	Species	Seasonality	Origin	November 2014	November 2016	April 2017	November 2017	Total
Andropogoneae	*Bothriochloa insculpta*	Annual	Indigenous	0	0	50 (1)	0	50 (1)
	*Chrysopogon zizanioides*	Perennial	Alien	0	10	0	0	10
	*Saccharum* spp.	Perennial	Alien	0	77	20	24	121
	*Sorghum arundinaceum*	Annual/perennial	Cryptogenic	17	102	97	73	289
	*Zea mays*	Annual	Alien	1	107 (14)	17 (9)	40 (8)	165 (31)
Bromeae	*Bromus catharticus*	Annual	Alien	0	4	0	0	4
Cynodonteae	*Chloris gayana*	Perennial	Alien	15	132	75	66	288
	*Chloris virgata*	Annual/perennial	Alien	0	24	22	22	68
	*Cynodon dactylon*	Perennial	Indigenous	12	134 (1)	27	53	226 (1)
	*Dactyloctenium*sp.	Annual	Indigenous	4	8	30	1	43
	*Eleusine indica*	Annual	Alien	5	22	4	54	85
Cypereae	*Cyperus rotundus*	Perennial	Cryptogenic	1	48	14	30	93
Eragrostideae	*Eragrostis*sp.	Annual/perrenial	Alien	0	50	65	69	184
Paniceae	*Brachiaria brizantha*	Perennial	Alien	0	0	0	2	2
	*Brachiaria decumbens*	Perennial	Alien	0	10	0	0	10
	*Brachiaria umbellata*	Perennial	Indigenous	1	15	51 (19)	62 (16)	129 (35)
	*Cenchrus echinatus*	Annual	Alien	1	19 (5)	34 (4)	38 (2)	92 (11)
	*Digitaria ciliaris*	Annual	Cryptogenic	27	99 (20)	5 (4)	18	149 (24)
	*Digitaria debilis*	Annual	Alien	0	0	1 (1)	0	1 (1)
	*Echinochloa colona*	Annual	Alien	0	0	3	0	3
	*Melinis repens*	Annual/perennial	Cryptogenic	21	76	95	61	253
	*Pennisetum clandestinum*	Perennial	Alien	0	7	0	0	7
	*Setaria pumila*	Annual	Alien	6	14	1	0	21
	*Megathyrsus maximus*	Perennial	Alien	32	142	115	80 (11)	369 (11)
	*Urochloa miliaceum*	Annual	Alien	0	0	0	32	32
	*Urochloa*sp.	Perennial	Cryptogenic	0	0	0	30	30
Paspaleae	*Paspalum dilatatum*	Perennial	Alien	1	6	17	18	42
	*Paspalum urvillei*	Perennial	Alien	0	0	0	1	1
Poeae	*Avena sativa*	Annual	Alien	0	84	0	10	94
Zoysieae	*Sporobolus africanus*	Perennial	Alien	0	6	1	16	23
Total				144	1,196 (40)	744 (38)	800 (37)	2,884 (115)

### Host identification

In cases where the species of a plant could not be determined by visual inspection, the species was identified using sequencing of the *matK* and *rbcL* genes as described previously (Charlery de la Masselière et al. 2017). Polymerase chain reaction (PCR) amplification was conducted before direct Sanger sequencing at Macrogen Europe (Netherlands). After a sequencing quality control step, sequences were classified using the Ribosomal Database Project (RDP) classifier (Wang et al. 2007) against a database of *matK* and *rbcL* plant sequences obtained from GenBank in August 2017.

### Total genomic DNA extraction

Two distinct DNA extraction procedures were used. In the first, the DNeasy Plant Mini Kit (Qiagen) was used according to the manufacturer’s instructions and DNA extracts were stored at −20°C before use. In a second procedure, total genomic DNA was extracted using a ‘leaf soak’ extraction protocol based on Roberts et al. (2000). Extracts were prepared from fresh leaf disc samples (obtained with one punch of a 200-µl PCR tube on a leaf) by adding 30µL of a solution containing 100 mM of Tris HCl, 1 M of KCl, 10 mM of ethylenediamine tetra acetic acid (pH 8.4), and an incubation at 95°C for 10 min. Leaf soak extracts were processed without storage. A total of 400 samples from the April 2017 sampling campaign were treated with both procedures and served as controls for the method (of the sample treated with both procedures, 92 per cent had similar infectious status and 96 per cent of the positive samples presented with the same main viral identification for the two processing methods, data not shown). Whereas the samples from the 2014 campaign were only treated using the DNeasy Plant Mini Kit, all other samples (from the November 2016 and November 2017 campaigns and the remaining samples from the April 2017 campaign) were treated with the sole leaf soak procedure.

### Eco-genomic approach

An eco-genomic approach based on rolling circle amplification (RCA) and random PCR amplification (RCA-RA-PCR) followed by high throughput sequencing was used as described in Claverie et al. (2019). Briefly, each DNA extract was subjected to RCA using the illustra Templiphi Kit (GE Healthcare) followed by a joint random amplification and tagging step. Importantly, tagging was performed in duplicate in distinct PCR runs and using distinct tags. In each batch of eighty samples of the April and November 2017 sampling campaign, a negative control (a DNA extract from a healthy tomato plant grown in the laboratory from seed) and a positive control (pUC19 plasmid provided as a positive control within the illustra Templiphi Kit) were included. After quantification of amplicons, as described in Claverie et al. (2019), up to 160 amplicons were pooled together before library construction. The pooling was performed with a maximum concentration ratio of 1.5 (i.e. no amplicons could have a concentration more than 1.5 times higher than any other amplicon within a single pool). Amplicon replicates for a given sample were sequenced within distinct pools. Pools were then purified using the illustra GFX PCR DNA and Gel Band Purification Kit (GE Healthcare) according to the manufacturer’s instructions before quantification and quality control using the Qubit dsDNA BR Assay Kit for the Qubit fluorometer (Thermo Fisher Scientific) and D5000 ScreenTape for 4200 TapeStation (Agilent Technologies). Library construction (including a Covaris shearing step) and Illumina sequencing (HiSeq2500 and 2x250pb paired-end sequencing for the November 2014 samples and HiSeqXten with 2x150pb PE sequencing for the other samples) were performed at Genewiz (USA). Up to fifteen libraries (corresponding to a maximum of 2,400 amplicons and 1,200 samples) were pooled per sequencing run. After a quality control step and removal of Illumina sequencing adapters using the processing pipeline described in Claverie et al. (2019), viral sequences were classified with similarity searches against both the viral RefSeq database (RefSeq release no. 84) using the BLASTx equivalent algorithm implemented in DIAMOND v0.9.19.120 (Tang et al. 2014) and a geminivirus and geminivirus-associated satellite (i.e. members of the *Alphasatellitidae* and *Tolecusatellitidae* families) database using BLASTn. Both databases were obtained from GenBank in October 2017. To reduce the dataset size, reads with similarities to geminivirus sequences were then clustered using SWARM v2.1.9 (Mahé et al. 2015) with the distance parameter set to three.

### Bioinformatic filters for sample classifications

Metagenomic projects are hindered by frequent cross-sample and cross-library contamination. Such contamination arises when nucleotide sequences from a given sample are incorrectly associated with some other sample. Cross-contamination can occur at distinct stages of the metagenomic procedure, such as during sample nucleic acid extraction, during sequencing library construction, during sequencing itself, or during downstream sequence processing and analysis. The nature of ultra-deep sequencing is such that even trace contaminants can be detected and therefore influence the outcomes of experiments (Tosar et al. 2014). It is therefore imperative to carefully evaluate and consider the possible cross-contamination of experiments to confidently identify the positive samples (Massart et al. 2017). To identify a series of read number thresholds to use to determine if a sample is likely infected, patterns of inter-sample contamination were monitored using the positive and negative controls within the sequencing run for the samples collected in April 2017.

Read analyses of the negative controls (DNA extracts of a healthy tomato plant grown in a climatic chamber) and the positive controls (pUC19-purified DNA) were performed: the number of geminivirus reads (which should have been absent from both controls) and the number of pUC19 reads (which should have been absent from the negative control and every field sample) were determined. The distribution of pUC19 reads was high in all the plasmid controls (a mean of 141,000 reads ranging from 32,000 to 299,000 reads). Conversely, a mean of seventeen pUC19 reads (ranging from 1 to 127 reads) was obtained per negative control, indicating inter-sample contamination (*y*-axis, Supplementary Fig. S1). Also indicative of cross-sample contamination, geminivirus reads were found associated with every control, with a mean of eighty-eight reads per control (*x*-axis, Supplementary Fig. S1, and more than 100 reads in seven out of the thirty controls). While this degree of cross-sample contamination might be considered high, it must be borne in mind that the metagenomic protocol involves an RCA amplification followed by a PCR procedure that would result in thousands of amplicons, making it unlikely that a successful PCR would only result in less than a few hundred virus reads for a genuinely virus-infected plant sample. Both the degree of contamination of plant samples with positive control–derived reads and the degree of contamination of controls with geminivirus reads are concordant in suggesting that a minimum of 100 reads from a particular virus taxon (i.e. greater than 1 per cent of the total reads obtained per sample) would be a conservative threshold above which a sample should be considered as likely being infected by that virus.

We defined four sample categories: (1) negative samples which included those for which >10,000 reads were obtained per replicate, of which fewer than 100 were detectably mastrevirus-derived; (2) positive samples (i.e. samples where >1,000 mastrevirus-derived reads were present in either of the two replicates or where both of the replicates contained >500 mastrevirus-derived reads); (3) doubtful samples (i.e. samples for which <500 mastrevirus reads were detected in either of the two replicates and where neither replicate had >1,000 mastrevirus-derived reads); and (4) failed samples (i.e. samples with <10,000 total reads for either of the two replicates).

### Mastrevirus taxonomic assignment and structure of plant–virus association

Sequences from positive samples were further classified using the phylogenetic placement approach implemented in pplacer (Matsen, Kodner, and Armbrust 2010) as described in Claverie et al. (2019). Placement was first performed against a database of all mastrevirus complete genome sequences available in GenBank in April 2018. For sequences classified as belonging to the species, MSV, in a first round of placement, another round of placement was performed against a database that included representatives of all MSV-strain complete genome sequences as obtained from GenBank in August 2019. Classifications were analysed using the ‘BoSSA’ R package (https://cran.r-project.org/package=BoSSA). Contingency matrices with the number of reads assigned to each viral species per sample were obtained after phylogenetic placement and positive-sample filtering. Samples with similar viral assignment profiles were grouped after the clustering based on the Kantorovich–Rubinstein distance matrices produced during the placement step using guppy v1.1 (Matsen, Kodner, and Armbrust 2010). Bipartite networks were generated using the ‘bipartite’ R package (Dormann et al. 2009).

### Full-genome sequencing

At least one sample from each viral group as defined after contingency table clustering was selected for full-genome cloning and sequencing. Full mastrevirus genomes were obtained using an RCA-restriction procedure as previously described in Inoue-Nagata et al. (2004). Briefly, 1 µl of RCA amplicon was digested using several enzymes (*Acc*I, *Bam*HI, *Eco*RI, *Kpn*I, *Nco*I, *Nde*I, *Pst*I, *Sac*I, *Sal*I, and *Sph*I) to identify those enzymes that cut only a single time with a monomeric viral genome sequence, i.e. to identify the restriction enzyme(s) that yielded a ∼2.7-kb fragment, the expected length of a mastrevirus genome. Concomitantly to the restriction digestions, 1 µl of RCA amplicon was amplified using back-to-back primers designed based on read alignments (Supplementary Table S1). Before the ligation to pJET 1.2 cloning vector (Thermo Fisher Scientific, USA), all fragments from restriction enzyme digests or PCR amplifications were purified using the illustra GFX PCR DNA and Gel Band Purification Kit (GE Healthcare, USA) according to the manufacturer’s instructions. The ligated products were then cloned in *Escherichia coli* (JM109, Promega), and the selected plasmids were purified using the QIAprep Spin Miniprep Kit (Qiagen) and sequenced by Macrogen Europe (Netherlands) using primer walking. Full-length mastrevirus genomes were assembled with Geneious v6.0.6 (Kearse et al. 2012). Full-nucleotide sequences were used as queries in BLAST searches of the National Center for Biotechnology Information nt database for preliminary species assignment. Pairwise similarity comparisons of full-nucleotide sequences of cloned genomes to the previously determined mastrevirus sequences with which they were most similar were performed using SDT v1.2 (Muhire, Varsani, and Martin 2014). All the mastrevirus sequences obtained during this study are available on GenBank under the accession numbers OQ211417–OQ211465.

### Phylogenetic and recombination analyses

Cloned sequences of each mastrevirus species along with all mastrevirus genomes previously characterised from Reunion and a selection of other AfSV sequences that were representative of the diversity of this viral group were aligned using MAFFT (Katoh and Standley 2013) after linearisation of all the sequences at the virion strand origin of replication. Maximum-likelihood phylogenetic trees were constructed using FastTree v2.1.8 (Price, Dehal, and Arkin 2010) and were edited using the ‘ape’ R package (Paradis et al. 2004). Recombination events were then detected within the alignment using the RDP (Martin and Rybicki 2000), GENECONV (Padidam, Sawyer, and Fauquet 1999), BOOTSCAN (Martin et al. 2005), MAXCHI (Maynard Smith 1992), CHIMERA (Posada and Crandall 2001), SISCAN (Gibbs, Armstrong, and Gibbs 2000), and 3SEQ (Boni, Posada, and Feldman 2007) methods implemented in the RDP5 program (Martin et al. 2021). As we were primarily interested in uncovering events that may have occurred in Reunion, rather than throughout the evolutionary history of mastrevirus diversification, a ‘group’ analysis was used in RDP5 so as to detect only recombination events involving sequences closely related to those detected on Reunion, i.e. the only triplets of sequences tested for recombination were those where the recombinant sequence was isolated on Reunion or sequences closely related to either of the recombinant’s two parental sequences were isolated on Reunion. All detected recombination events were then manually inspected and filtered to identify those recombination events that were likely to have occurred on Reunion in the fairly recent past. Besides the grouping scheme, default RDP5 settings were used and recombination events were considered significant when detected by at least three different recombination detection methods.

## Results and discussion

### Poales diversity within a small agro-ecosystem in Reunion

An ‘eco-genomic’ approach was used to describe the diversity and distribution of mastreviruses within a ∼17,000-m^2^ plot on the island of Reunion. This entailed sampling 2,884 individual plants collectively representing thirty Poales species. It must be noted here that sampling locations within the plots were not recorded and it is plausible that, in a small number of instances, an individual perennial plant may have been sampled in different surveys. In all, 86 per cent of the samples were uncultivated grasses (weeds and wild plants (2,472/2,884), twenty-six species; Table 1) and 14 per cent were from four cultivated grass species (412/2,884). Only 4 per cent of the samples (115/2,884) displayed visible streak symptoms. The sampled plants included fourteen perennial species (1,351/2,884; 47 per cent of the samples), four annual/perennial species (i.e. plants that can behave as annual or perennial depending on growing conditions; 794/2,884; 28 per cent of the samples), and twelve annual species (739/2,884; 25 per cent of the samples; Table 1). Twenty-one of the sampled plant species were alien to Reunion (56 per cent, 1,622/2,884), four were indigenous (16 per cent, 448/2,886), and five were cryptogenic (i.e. of unknown origin, 28 per cent, 814/2,884; Table 1). It is interesting to notice the high frequency of alien plants, a frequent characteristic of tropical Islands where native ecosystems have been heavily transformed (Macdonald et al. 1991).

### Bioinformatics filters and classification of infection status

**Figure 1.**
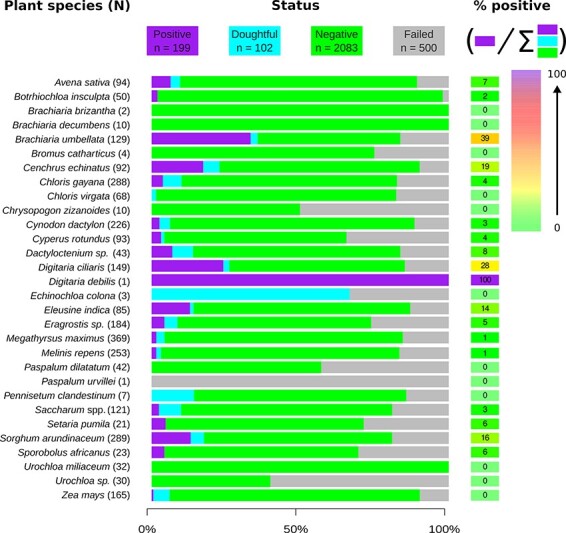
Infection status and infection rates for each Poales species. The number in brackets besides species names refers to the number of collected samples. Histogram colours refer to the infection status with positives in purple, doubtful in light blue, negatives in green, and unknowns in grey. The proportion of infected samples for each species is indicated in the cell on the right where the colour of the cell is set according to the infection rate (see the scale on the top right).

Based on the defined bioinformatic filters (see the Materials and methods section), 17 per cent (500/2,884) of the samples were discarded from the analysis as having too few raw sequencing reads for at least one of the two replicate sequencing runs carried out on each sample (i.e. sample for which an ‘infected’ status could not be confidently assigned to the sample; see the ‘failed’ category in Fig. 1 and Supplementary Table S2). Of the remaining 2,384 samples, 199 (8.3 per cent) were confidently identified as being infected by a mastrevirus. A further 102 samples (4.3 per cent) had low numbers of associated sequence reads that may have been derived from mastreviruses but were considered as negative because the read numbers could not be confidently differentiated from background contamination (see the Materials and methods section). If these doubtful samples were to be considered as infected by a mastrevirus, it would increase the prevalence to 12.6 per cent (Fig. 1; Supplementary Table S2). Erring on the side of conservatism, these 102 samples were not considered in further analyses.

In previous plant eco-genomic studies, Muthukumar et al. (2009), Roossinck et al. (2010), and Bernardo et al. (2018) found global viruses prevalence of 25, 70, and 15.6 per cent, respectively. In Roossinck et al. (2010) and Bernardo et al. (2018), the mean prevalences at the family level (the most precise taxonomic levels for viruses in these two studies) were of 6.4 and 0.9 per cent, respectively. In our study, the prevalence rate of 8.3 per cent for the *Mastrevirus* genus (and the Geminiviridae family in general, since (1) no other geminiviruses were discovered and (2) our protocol is suitable for all ssDNA viruses) is high but consistent with previous findings.

### Uncultivated, annual, or indigenous grasses tend to have higher mastrevirus infection frequencies than cultivated, perennial, or exotic grasses

Among the thirty Poales species that were collected, 63 per cent (nineteen/thirty) were identified as mastrevirus hosts. These species include three that are cultivated (ten samples) and sixteen that are uncultivated (189 samples) (Fig. 1; Supplementary Table S2). The frequencies of mastrevirus infection were significantly lower (*P*-value = 4.5 × 10^−5^) for the cultivated species (2.7 per cent, 10/363) than they were for the uncultivated species (9.3 per cent, 189/1832).

The infection rates of annual (11.6 per cent, 76/655) plants were significantly higher (*P*-value = 1.5 × 10^−3^) than those of perennial plant species (6.7 per cent, 73/1,092). The infection frequencies of plant species that were not easily classified as being annuals or perennials (7.8 per cent, 50/637) were intermediate between, and not significantly different from those of the annual or perennial plants.

The infection rates of exotic species (4.8 per cent, 65/1,346) were significantly lower (*P*-values <1 × 10^−6^) than those of both cryptogenic species (12.5 per cent, 81/645) and indigenous species (13.5 per cent, 53/393). The infection rates of cryptogenic and indigenous species were not significantly different.

Of the sampled species with sample sizes of greater than twenty, *Brachiaria umbellata* had the highest infection rate (39 per cent), followed by *Digitaria ciliaris* (28 per cent), *Cenchrus echinatus* (19 per cent), *Sorghum arundinaceum* (16 per cent), and *Eleusine indica* (14 per cent). All five of these species are uncultivated.

### The prevalence and host ranges of different mastrevirus species

**Figure 2.**
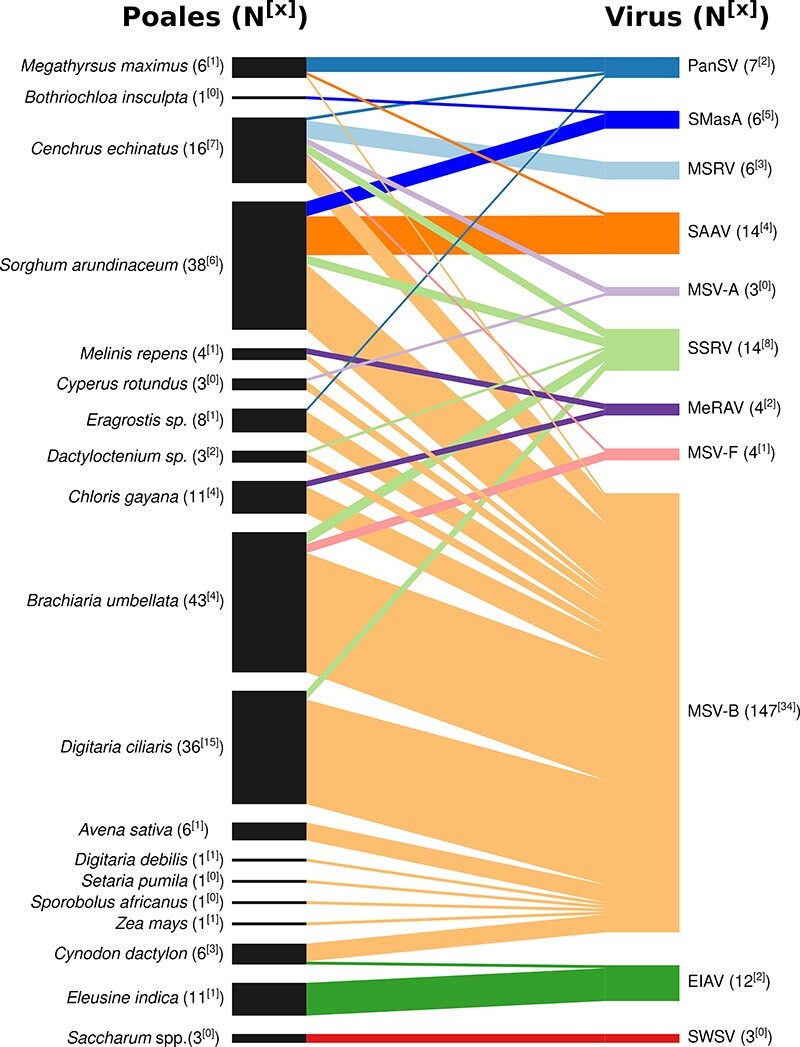
The Bipartite interaction graph representing the association between plant species (left side of the diagram) and viral species and strains (right side of the diagram) within the size of the boxes and links proportional to the number of positive plant samples. Numbers in brackets indicate the number of samples, and numbers in square brackets indicate the number of samples with cloned virus sequences.

The mastreviruses infecting the analysed plants belonged to eight different species (Fig. 2; Supplementary Table S3), four of which have been previously observed both in Reunion and elsewhere (MSV, maize streak Reunion virus (MSRV), sugarcane streak Reunion virus (SSRV), and sugarcane white streak virus (SWSV)). Three additional mastrevirus species, *Eleusine indica*–associated virus (EIAV), *Melinis repens*–associated virus (MeRAV), and *Sorghum arundinaceaum*–associated virus (SAAV), have only previously been detected in Reunion (Claverie et al. 2019). Panicum streak virus (PanSV), which has never previously been found in Reunion, was also detected. Other than for SWSV, we confirmed the presence of these various species with full-genome cloning and sequencing.

#### Maize streak virus

The most prevalent species was MSV with three strains identified: (1) MSV-A in three samples; (2) MSV-B in 147 samples (confirmed with thirty-seven full-genome sequences); and (3) MSV-F in four samples (confirmed with one full-genome sequence; Fig. 2; Supplementary Table S3). It is noteworthy that this is the first detection of the MSV-F strain in Reunion. It was found to infect four samples of an uncultivated plant species (three *B. umbellata* samples and one *C. echinatus* sample). MSV-F has previously been detected in Burundi, Nigeria, Uganda, Zimbabwe, and Mauritius and exclusively on uncultivated plants.

MSV-A sequence reads were detected in three samples, but we were unable to confirm the occurrence of complete MSV-A genomes within the relevant plant samples with full-genome cloning and sequencing. The absence of a genome sequence therefore makes it difficult to determine whether MSV-A is actually present in the samples, or if the reads assigned to MSV-A are traces of the introgression through genetic recombination of MSV-A-derived sequence fragments into another MSV-strain or another mastrevirus species. Indeed, below we confirm the presence of recombinationally acquired MSV-A-derived sequences within the genomes of different MSV-B lineages.

If the plants in which MSV-A sequences were detected are actually infected with MSV-A, the low prevalence of this MSV strain in Reunion (a maximum of three samples for MSV-A, compared to 147 samples for MSV-B) is unexpected. MSV-A has been extensively described in Africa and the SWIO Islands infecting maize, sugarcane, and numerous uncultivated plant species (Varsani et al. 2008; Kraberger et al. 2017). MSV-A causes MSD and is the most economically important mastrevirus lineage. It has a large geographical distribution in Africa, and it has been repeatedly found on various Indian Ocean Islands including Madagascar, Comoros, Mauritius, and Reunion. Whereas the first reports of MSD in Reunion date to the 1930s (Kopp 1930; Kopp and D’emmerez de Charmoy 1931, 1932), the first definitive reports of MSV were in the 1970s (Baudin 1976; Delpuech et al. 1986) and the first MSV-A clone was sequenced in the 1990s by Peterschmitt et al. (1996). However, because of the high impact of severe disease associated with MSV-A on maize crops, maize varieties resistant to MSV-A were selected in Reunion from a tropical composite population resistant to streak disease in the 1990s (Pernet et al. 1999a,b). The rapid diffusion of these resistant varieties across the island has led to a rapid decrease in the prevalence of MSD to the point where MSD symptoms are no longer reported on the island. The low prevalence of MSV-A in the studied agro-ecosystems may be associated with the global diffusion of resistant maize cultivars limiting the spread and maintenance of MSV-A in the environment in the absence of sensitive maize genotypes.

#### Maize streak Reunion virus

MSRV was detected in six plants exclusively from the uncultivated grass species,*C. echinatus.* This detection was confirmed with the cloning and sequencing of one full genome. The complete genome was most closely related to the isolates already obtained in Reunion. MSRV has previously been identified infecting both maize and uncultivated plants in Nigeria (*Setaria barbata* and *Rottboellia*sp.) and Reunion (*C. echinatus*) and maize in Ethiopia and China (Hadfield et al. 2012; Oluwafemi et al. 2014; Chen et al. 2015; Claverie et al. 2019; Guadie et al. 2019).

#### Sugarcane streak Reunion virus

SSRV was detected in fourteen samples from five uncultivated species (*B. umbellata, C. echinatus, Dactyloctenium*sp., *D. ciliaris*, and *S. arundinaceum*), and the detection was confirmed with the cloning and sequencing of eight full-genome sequences, sharing between 99 per cent identity with isolates previously obtained in Reunion (Fig. 2; Supplementary Table S3). SSRV has been previously described infecting both sugarcane and uncultivated species in Reunion (*S. barbata*) and uncultivated species in Nigeria (*Eleusine coracana*) and Zimbabwe (*Paspalum conjugatum*; Bigarré et al. 1999; Oluwafemi et al. 2008; Shepherd et al. 2008; Varsani et al. 2008; Kraberger et al. 2017).

#### Sugarcane white streak virus

SWSV was detected in three samples of sugarcane but was only confirmed through the cloning of a partial genome. Interestingly, SWSV is a recently discovered mastrevirus that has been described in Reunion, Egypt, Sudan, and Barbados (Candresse et al. 2014; Boukari et al. 2017; Claverie et al. 2019). It is thought that its worldwide spread was achieved through sugarcane transfers, while this virus was not diagnosed by quarantine controls before its discovery in 2014. The absence of clear symptoms and the failures of conventional virus detection methods to determine plant health status were apparently key to its diffusion.

#### Other mastrevirus species and a mastrevirus-associated alphasatellite

The three mastrevirus species previously found only in Reunion, EIAV, MeRAV, and SAAV (Claverie et al. 2019), all of which were in fact initially described from samples collected during the November 2014 survey described here, were all detected across samples taken in multiple years (EIAV in November 2014, 2016, and 2017; MeRAV in November 2014 and 2016; and SAAV in November 2014 and 2016 and April 2017; Supplementary Table S3). Based on read classifications, twelve samples were detectably infected with EIAV, four with MeRAV, and fourteen with SAAV. EIAV was identified in *Cynodon dactylon* and *E. indica*, MeRAV in *C. gayana* and *M. repens*, and SAAV in *S. arundinaceum* and *Megathyrsus maximus* plants (Fig. 2; Supplementary Table S3). Two complete EIAV genomes, two complete MeRAV genomes, and four complete SAAV genomes were cloned and sequenced (Fig. 2; Supplementary Table S3). Intra-species diversity was low with pairwise genome sequence identities being equal to or greater than 99.0, 99.5, and 98.8 per cent for EIAV, MeRAV, and SAAV, respectively. In EIAV, two indels of thirty-six nucleotides in the *mp* gene (at Position 415/416 relative to MK546379) and of ninety-five nucleotides in the *rep* gene (at Position 2122/2123 relative to MK546379) differentiate the two isolates.

The PanSV sequences identified here represent the first detection of this mastrevirus species in Reunion. Among the SWIO Islands, PanSV has only been previously detected in Mayotte (PanSV-G). In total, seven plant samples (five *M. maximus* and one each of *C. echinatus* and *Eragrostis*sp.) were found to be infected by PanSV. The presence of the virus was confirmed by sequencing two full genomes from two samples (Fig. 2; Supplementary Table S3). Considering the 94 per cent strain demarcation level for mastreviruses (Muhire et al. 2013), the Reunion isolates of PanSV correspond to a new strain provisionally named PanSV-J (it has 91.6 per cent identity with its nearest relative, PanSV-H from Nigeria, KM229918). The discovery of this new PanSV strain expands the known geographical distribution of this mastrevirus species and challenges the assertion that PanSV might have a more restricted geographical distribution than MSV (Kraberger et al. 2017).

Finally, a Sorghum mastrevirus–associated alphasatellite (SMasA; first described in Claverie et al. 2020) was detected in six samples (five *S. arundinaceaum* and one *Botriochloa insculpta*). Whereas no mastreviruses were detected in five of the samples, the alphasatellite was detected in a coinfection with SAAV in one *S. arundinaceum* plant.

#### *Structure of*Mastrevirus*–host species interaction networks suggests that most mastrevirus species are generalists*

Given that, with no known exceptions, mastreviruses are leafhopper transmitted, the distribution of any particular virus species among plants within the analysed agro-ecosystem will reflect, at least in part, the movement characteristics and feeding preferences of their specific leafhopper vectors (Elena, Fraile, and García-Arenal 2014). The host range of a vector-transmitted virus is necessarily limited by the vector’s feeding preferences (Power 2008). Therefore, any expansion of the host range of the vector could result in an expansion of the host range of viruses transmitted by that vector (Harrison and Robinson 1999). MSV (Nielson 1968), PanSV (Briddon et al. 1992), SSRV (Bigarré et al. 1999), and SSV (Briddon et al. 1996) are all known to be transmitted by leafhopper species in the genus *Cicadulina* (Shepherd et al. 2010), with *C. mbila* Naudé (which is abundantly found in Reunion, Bonfils, Quilici, and Reynaud 1994) being one of the most important vector species (Webb 1987). The vector species of EIAV, MeRAV, MSRV, SAAV, and SWSV remain unknown. It should therefore be borne in mind that, while likely impacted by vector behaviour, our analysis of mastrevirus–host ranges was restricted to plant–virus interactions and that the host species in which particular virus species were detected—the minimal host range—are likely not the only host species among those considered that these viral species can infect.

A second potentially important limitation of our study lies in our global sampling scheme. Whereas we construct our plant–virus infection network with the accumulation of data collected over multiple sampling campaigns, neither the timings of the collections (here, the month of collection being either April or November) nor the precise locations of the sampled plants (i.e. the habitat, such as cultivated field or fallow) were taken into account. Additionally, as a single location was sampled during the survey, it is entirely possible that a different interaction network would be evident in another place. It is therefore possible that the global network is a biased representation of the infection networks that could exist at a certain time in a certain place and that the actual structure of the network might either evolve through the year or be dependent on the specific habitat (Valverde et al. 2020). We would therefore consider the network that we have presented to represent the overall minimal host ranges of mastreviruses.

The overall minimal host ranges of the mastreviruses uncovered during the study were represented as a bipartite network (Fig. 2). In this representation, virus species are connected to the plant species they were found infecting. Out of the nineteen Poales species infected by mastreviruses, sixteen were hosts of MSV-B (Fig. 2). Besides SWSV and MSRV which were each found in only one Poaceae species, the other seven mastreviruses (EIAV, MeRAV, MSV-A, MSV-F, PanSV, SAAV, and SSRV) were each found in two or more plant species (Fig. 2).

While this pattern might be taken as suggestive of SWSV and MSRV being ‘specialist’ viruses that are narrowly adapted to infecting one or a small number of host species, one should be cautious when coming to this conclusion. Only small numbers of plants were found infected with these viruses: six for MSRV and three for SWSV.

Conversely, the detection of all the other mastrevirus species in multiple hosts may suggest that these viruses are likely generalists (Elena, Agudelo-Romero, and Lalic 2009) and able to infect, replicate in, and get transmitted from multiple different host species. However, there is no definitive threshold denoting the number of distinct host species a virus must be able to infect for it to be considered as a generalist: there is in fact a continuum of ‘generalistness’ with an extreme-generalist virus such as cucumber mosaic virus (CMV) being capable of infecting over 1,000 plant species in multiple different families (including monocots and dicots; Edwardson and Christie 1991). A more moderately generalist virus could be exemplified by MSV, which is capable of infecting over 100 plant species, albeit all of which are restricted to a single family, the Poaceae (Damsteegt 1978). It must be noticed however that the detection in this study of MSV from three plants of *Cyperus rotundus* from the Cyperaceae family would extend the host range to another plant family if these were further confirmed. Indeed, we found that the most prevalent MSV strain detected in our study, MSV-B, was found infecting plants belonging to sixteen species.

Although less obviously generalist, the fact that the other mastreviruses detected here were found in multiple host species in the very restricted survey area suggests that these too may be generalists: albeit not as generalist as MSV. Even though generalist viruses have more opportunities for transmission as a result of their larger host ranges (Woolhouse, Taylor, and Haydon 2001), due to the assumption of the adaptive trade-off needed to enable the infection of multiple host species, the theory suggests that natural selection will favour specialisation over generalism (Futuyma and Moreno 1988). Indeed, experiments in homogenous environments such as those created in the laboratory have indicated that the fitness of generalists tends (but is not guaranteed) to be lower than that of specialists (Kawecki 1994; Bedhomme, Hillung, and Elena 2015). One likely reason for this relates to the compactness of viral genomes. In compact genomes, the fitness value of a mutation at one site will frequently be contingent on mutations occurring at other genome sites—a phenomenon referred to as epistasis. Furthermore, it is expected that in some cases adaptive mutations that increase viral fitness in one host (i.e. mutations that directly or indirectly increase probabilities of onward transmission in this host) will be maladaptive and decrease fitness in another host: a phenomenon referred to as antagonistic pleiotropy (Whitlock 1996; Moury and Simon 2011; Remold 2012). It is, however, not guaranteed that adaptive mutations in one host will have fitness-reducing consequences in other hosts. Instead, adaptive mutations in one host might also be adaptive in others and some generalists might be more accurately described as jacks of all trades and masters of all: or, more formally, as no-cost generalists (Remold 2012). Many emerging viruses have been found to be generalists including tomato yellow leaf curl virus (family Geminiviridae; Yan et al. 2021), CMV (family *Bromoviridae*; Edwardson and Christie 1991), and chickpea chlorotic dwarf virus (family Geminiviridae; Kraberger et al. 2015). This suggests that environmental heterogeneity and variations in host availability across landscapes and over time—environmental factors likely to favour the evolution of generalists—may be key in the emergence of novel pathogens (Fraile et al. 2017; McLeish, Fraile, and García-Arenal 2018).

### Many Poales species either potentially, or do, host coinfections of genetically divergent mastreviruses

**Figure 3.**
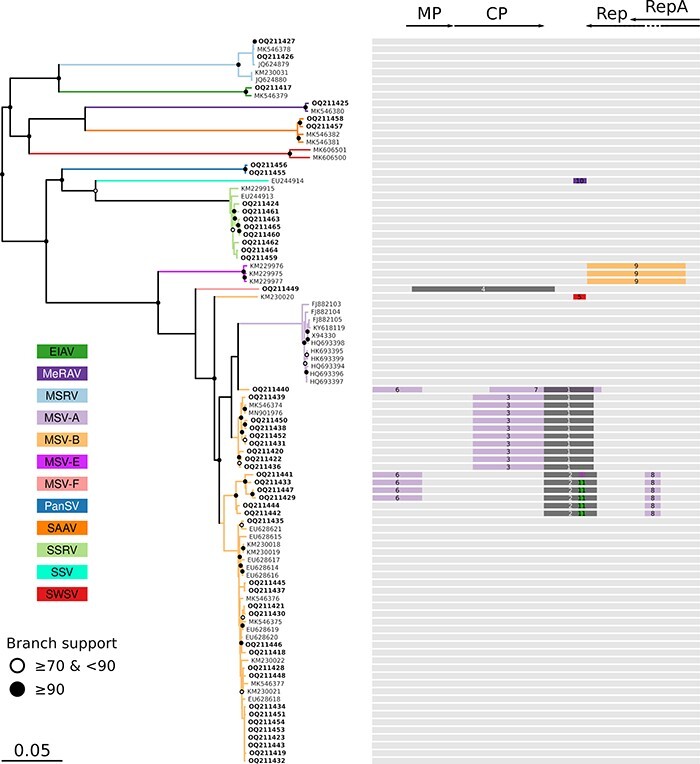
Phylogenetic relationships and recombination patterns among the AfSV species sampled in Reunion. The neighbour-joining tree contains thirty-five complete genomes of monocot-infecting mastreviruses from Reunion and fifty-nine complete genomes determined in this study (indicated in the bold font). Branches and tip labels are coloured according to the mastreviruses species and strains. Open and closed circles on nodes indicate bootstrap support for the branches to their left of 70–89 and ≥90 per cent, respectively. Recombination events detected using RDP5 are depicted with tracks coloured depending on the minor parent species/strains (unknown origins are depicted with the dark grey) over a linear schematic representation of the viral genome. Arrows and blocks at the top correspond to open reading frames: movement protein (MP), coat protein (CP), and replication-associated proteins (Rep and Rep A).

Most of the analysed Poales species (eleven/nineteen) are hosts for more than two of the detected mastrevirus species (Figs 2 and 3). More importantly, four plant species were found to be hosts for more than three mastrevirus species. These plants, which might be considered as ‘hub’ or ‘cornerstone’ mastrevirus hosts (Fig. 3), are *C. echinatus*, which hosts four of the identified mastrevirus species, and *C. gayana, M. maximus*, and *S. arundinaceum*, which each host three of the mastrevirus species. Among these hosts, only *C. echinatus* is an annual species with all the others, but *S. arundinaceum* is definitively perennial (Table 1). What this means is that individual plants of these species can likely remain persistently infected with multiple different mastrevirus species for multiple years: a characteristic that is likely to foster frequent instances of coinfections of individual plants with evolutionarily divergent mastreviruses.

Altogether, twenty coinfections with different mastrevirus species and satellites were detected among the 199 mastrevirus-infected plant samples, including one coinfection with SAAV and SMasA (Supplementary Table S4; Claverie et al. 2020). Five additional coinfections were detected that involved different MSV strains (three MSV-A/MSV-B and two MSV-B/MSV-F coinfections). The coinfections were confirmed via cloning and Sanger sequencing from four samples (*B. umbellata, C. echinatus*, and *D. ciliaris*). It is apparent, therefore, that not only can these hub plant species host different mastreviruses for multiple years at a time, but they may also be simultaneously coinfected with either multiple different mastrevirus species or multiple genetically distinct strains of one species: a factor that is likely to both foster the occurrence of genetic recombination and promote the selection over time of recombinants with recombination breakpoint patterns that minimally disrupt, and/or maximally optimise, combinations of fitness-enhancing genetic polymorphisms (van der Walt et al. 2009; Lefeuvre and Moriones 2015; Jammes et al. 2022).

### Inter-strain and inter-species recombination among Reunion mastreviruses

It is well known that intra- and inter-species recombination is common in mastreviruses (Varsani et al. 2008). A good example is the MSV-A strain, which is the likely descendant of a recombinant virus with MSV-B and MSV-G/F parents (Varsani et al. 2008).

**Figure 4.**
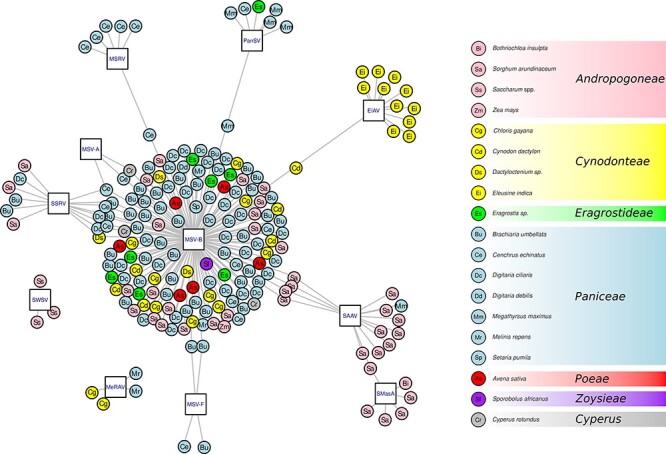
An interaction network representing the associations between plant samples (circles) and viral species/strains (squares). For each plant sample, the acronym indicates the plant species and the colour is set according to the plant tribe (see the legend on the right side of the figure). The samples are linked using grey edges to the virus group whenever an infection was definitively detected.

In order to assess the extent of recombination within mastreviruses sampled from Reunion, we performed a recombination analysis on all the full-genome sequences obtained in this study along with sequences representative of all known AfSV species and strains. Importantly, the analysis was performed so that only events considered were those involving Reunion sequences as recombinants with at least one of the identified parental sequence lineages having also been detected on Reunion. Eleven recombination events of this type were detected. Nine of these (all except Events 4 and 9) only involved viral lineages previously detected on Reunion and may have plausibly occurred on the island (Table 2). Five events were identified as intra-species (or inter-strain) recombination, all between different MSV lineages: inter-strain recombinants were between MSV-B and -A (Events 3, 6, 7, and 8; Table 2; Fig. 4) and MSV-F and -B (Event 9). Interestingly, whereas one of the MSV inter-strain recombination events was detected in sequences isolated in previous studies (dating back to 2006, Event 9), the other inter-species recombination events were only detected in sequences isolated during this study.

**Table 2.** The list of recombinant events detected within the mastreviruses sequences obtained from Reunion samples.

Event ID	Begin^a^	End^a^	No. of sequences	RUN only^b^	Recombinant sequence(s)	Minor parental sequence(s)	Major parental sequence(s)	Min. *P*-value	Method^c^
1	1,202	1,403	11	Yes	MSV-B (OQ211436)	Unknown (most similar to MSV-A)	MSV-B	2.91e-29	RG**B**MCS3
2	1,202	1,425	6	Yes	MSV-B (OQ211444)	Unknown (most similar to MSV-B)	MSV-B	1.83e-26	RG**B**MCS3
3	590	1,202	10	Yes	MSV-B (OQ211436)	MSV-A (KY618119; 99.7%)	MSV-B	5.33e-23	RG**B**MCS3
4	179	1,230	9	No	MSV-B (OQ211449)	Unknown (most similar to MSV-B)	MSV-B	1.01e-22	RGBMCS**3**
5	1,277	1,351	1	Yes	MSV-B (KM230020)	SWSV (MK606500; 80.8%)	MSV-B	5.38-15	**R**GBM3
6	2,648	262	5	Yes	MSV-B (OQ211441)	MSV-A (KY618119; 99.3%)	MSV-B	1.32e-12	RG**B**MCS3
7	768	1,482	1	Yes	MSV-B (OQ211440)	MSV-A (FJ882105; 99.8%)	MSV-B	1.13e-8	R**G**BMCS
8	1,800	1,879	6	Yes	MSV-B (OQ211442)	MSV-A (KY618119; 98.7%)	MSV-B	1.66e-8	**R**GB3
9	1,356	2,085	10	No	MSV-E (KM229977)	MSV-B (KM230022; 92.5%)	Unknown (most similar to MSV-A)	8.55e-8	R**M**C3
10	1,305	1,382	1	Yes	SSV (EU244914)	MeRAV (RE525_20h; 76.5%)	SSV	1.30e-7	**R**GBM3
11	1,305	1,342	6	Yes	MSV-B (OQ211429)	EIAV (MK546379; 100%)	MSV-B	2.64e-6	RGMC**S**3

a Positions are relative to the recombinant sequence indicated in parenthesis in the ‘Recombinant sequence(s)’ column.

b This indicates if events were specific to sequences from Reunion.

c Detection methods: RDP (R), GENCONV (G), BOOTSCAN (B), MAXCHI (M), CHIMERA (C), SISCAN (S), and 3SEQ (T). The method with the highest *P*-value for each recombination event is shown in bold.

Three of the detected recombination events were identified as inter-species events (Events 5, 10, and 11). Whereas two of these recombination events involve sequences distantly related to isolates previously identified on Reunion, the clearest signal of inter-species recombination (Event 11) that may have plausibly occurred on Reunion Island was observed in a clade of predominantly MSV-B-like sequences that possessed a sequence tract derived from a parental virus most closely resembling EIAV.

## Concluding remarks

Globally, our study reveals the breadth and abundance of mastreviruses infecting cultivated and uncultivated plants within the context of a small agro-ecological landscape. Eleven virus species and genetically diverse strains of some of these were found infecting nineteen different plant species. The host ranges of these species, depicted as a virus–host association network, revealed a high degree of structure despite indications that several of the mastrevirus species are likely generalists with multiple different natural hosts.

Globally, a greater infection rate was found among uncultivated hosts than was found among cultivated hosts, which superficially contradicts previous discoveries of infection rates increasing with host density (Agrawal, Lau, and Hambäck 2006; Keesing et al. 2010). It must be noted, however, that whereas uncultivated plants were abundantly present across the analysed area and throughout the year-long study period, cultivated plants were spatially clustered when present and were periodically absent between cropping seasons. The intermittent presence within the study area of cultivated species such as oats and millet that are infrequently grown in Reunion may make it less likely that viruses on the island would have had sufficient opportunities to adapt to optimally use these plant species as hosts.

Whereas temporal variations in biotic and abiotic conditions may leave a strong imprint on host–virus interaction networks (Valverde et al. 2020), the structure of such networks will in turn likely influence patterns of virus evolution. Specifically, in Reunion perennial plant species such as *C. gayana, M. maximus*, and *S. arundinaceum* that can host multiple different genetically divergent mastreviruses are far more likely to be the sites of mixed mastrevirus infections and inter-species or inter-strain recombination events than annual plants belonging to species that host only a single mastrevirus strain or species. More generally, our findings are consistent with the hypothesis that perennial hosts sitting at the hubs of virus–host interaction networks are likely to play a disproportionately large role in fostering the evolution and emergence of new recombinant mastrevirus variants, some of which might be plausibly anticipated to have substantially altered transmission and pathogenicity phenotypes relative to their parents (Varsani et al. 2008; van der Walt et al. 2009).

While the ecological importance of these perennial ‘hub’ hosts with respect to the transmission dynamics and persistence of mastrevirus species over decades-long time periods might appear obvious, it remains to be tested what would happen if these hosts were removed from virus–host interaction networks. Would the networks collapse and inter-species or inter-strain recombination rates diminish, or might some of the viral species or strains go extinct? These are difficult questions to answer using surveys of the sort described here, but the unexpectedly low prevalence of MSV-A within the plants that we sampled may provide a clue as to how this could be experimentally tested. Specifically, the impact on viral prevalence and virus–host interaction networks of removing a key host species could be determined on other Indian Ocean Islands simply by introducing highly MSV-resistant maize genotypes and performing the same sort of surveys throughout this process as those described here. Furthermore, future periodic surveys on the same sampling site used in our study would certainly reveal whether the currently observed viral prevalence and host-range patterns vary widely over time or whether they are robustly maintained over decades-long time frames.

## Supplementary Material

vead043_Supp

## Data Availability

Sequences described in this study are available on GenBank under the accession numbers OQ211417–OQ211465.
